# Systems genetics reveals the influence of expression QTLs in mouse embryonic stem cells on transcriptional variation later in differentiated neural progenitor cells

**DOI:** 10.1093/g3journal/jkaf099

**Published:** 2025-05-06

**Authors:** Selcan Aydin, Daniel A Skelly, Hannah B Dewey, J Matthew Mahoney, Ted Choi, Laura G Reinholdt, Christopher L Baker, Steven C Munger

**Affiliations:** The Jackson Laboratory, Bar Harbor, ME 04609, USA; The Jackson Laboratory, Bar Harbor, ME 04609, USA; The Jackson Laboratory, Bar Harbor, ME 04609, USA; Graduate School of Biomedical Sciences, Tufts University, Boston, MA 02111, USA; The Jackson Laboratory, Bar Harbor, ME 04609, USA; Predictive Biology, Inc., Carlsbad, CA 92010, USA; The Jackson Laboratory, Bar Harbor, ME 04609, USA; Graduate School of Biomedical Sciences, Tufts University, Boston, MA 02111, USA; The Jackson Laboratory, Bar Harbor, ME 04609, USA; Graduate School of Biomedical Sciences, Tufts University, Boston, MA 02111, USA; The Jackson Laboratory, Bar Harbor, ME 04609, USA; Graduate School of Biomedical Sciences, Tufts University, Boston, MA 02111, USA

**Keywords:** genetic diversity, eQTL, systems genetics, neural progenitor cells, mediation analysis

## Abstract

Genetic variation leads to phenotypic variability in pluripotent stem cells that presents challenges for regenerative medicine. Although recent studies have investigated the impact of genetic variation on pluripotency maintenance and differentiation capacity, less is known about how genetic variants affecting the pluripotent state influence gene regulation later in development. Here, we characterized expression of 12,000 genes in a large panel of donor-matched Diversity Outbred mouse embryonic stem cell and mouse neural progenitor cell lines. QTL mapping identified 4,060 expression QTLs in mouse neural progenitor cells, including 2,998 local and 1,062 distant expression QTLs. In a comparison of mouse neural progenitor cell and mouse embryonic stem cell expression QTLs, we found that local expression QTLs were more likely than distant expression QTL to be detected in both cell types. Distant expression QTLs were largely unique to 1 cell type, and we mapped 3 mouse neural progenitor cell–specific expression QTL hotspots on chromosomes 1, 10, and 11. Mediation analysis of the chromosome 1 hotspot identified *Rnf152* as the best candidate mediator expressed in mouse neural progenitor cells, while cross-cell-type mediation using mouse embryonic stem cell gene expression along with partial correlation analysis strongly implicated genetic variant(s) affecting *Pign* expression in the mouse embryonic stem cell state as regulating the mouse neural progenitor cell chromosome 1 hotspot. These findings highlight that local mouse neural progenitor cell expression QTLs are more likely than distant expression QTLs to be shared with mouse embryonic stem cells; distant mouse neural progenitor cell expression QTLs are numerous but largely unique to that cell type, with many colocalizing to mouse neural progenitor cell–specific hotspots; and mediation analysis across cell types suggests that expression of *Pign* in mouse embryonic stem cells shapes the transcriptome of the more specialized mouse neural progenitor cell state.

## Introduction

Pluripotent stem cells (PSCs) have the capacity to self-renew indefinitely in culture and differentiate into any cell type. These properties make them a valuable resource for the study of gene regulation in various cellular contexts, giving us access to cell types that are otherwise difficult to obtain or follow through developmental time. Recently, systems genetics approaches have combined the power of genetic diversity with the differentiation potential of PSCs to identify genetic variants that influence gene expression regulation across different cellular contexts (Warren and Cowan 2018; Swanzey *et al*. 2021; Farbehi *et al*. 2024). For example, mouse embryonic stem cell lines (mESCs) derived from diverse strains have been used to study the maintenance of the pluripotent state, early lineage specification, and the stability of genomic imprinting (Skelly *et al*. 2020; Byers *et al*. 2022; Aydin *et al*. 2023; Parikh et al. 2025). Similarly, the influence of genetic variation on human induced PSCs (iPSCs) has been characterized at multiple molecular levels (Kilpinen *et al*. 2017; Panopoulos *et al*. 2017; Banovich *et al*. 2018; Mirauta *et al*. 2020) and throughout differentiation into various cell types (Cuomo *et al*. 2020; Jerber *et al*. 2021; Elorbany *et al*. 2022; Wells *et al*. 2023; Popp *et al*. 2024). Despite these examples, our understanding of the downstream consequences of pluripotent state variation on developmental trajectories and later stages of development remains largely undefined. To address this gap, we turned to a system where we could conduct systems genetics inquiry in both the pluripotent and a subsequent developmental stage using cells from the same donors.

PSCs can be differentiated into neural progenitor cells (NPCs) that can proliferate in culture continuously and have the capacity to further differentiate into specialized neuronal cell types such as neurons and astrocytes (Conti *et al*. 2005). Similar to their pluripotent ESC progenitors, NPCs represent a dynamic cell type maintained through external signaling molecules and growth factors in cell culture rather than a distinct cell type observed in vivo. NPCs are an increasingly important resource for cellular modeling of neurodevelopment and neurodegenerative disorders. For example, Young-Pearse and colleagues recently analyzed neuronal cell types differentiated from a set of 50 iPSC lines from the Religious Orders Study and Memory and Aging Project aging cohorts to identify a neuron-specific interaction between the Alzheimer's disease risk genes SORL1, APOE, and CLU (Lee *et al*. 2023). In a separate study, genetically diverse NPCs derived from human iPSCs were recently used to identify the genetic variants driving differences in susceptibility to Zika virus (Wells *et al*. 2023). Finally, another recent study profiled hPSC differentiation to mature neurons with single-cell RNA sequencing (scRNA-seq) across a fine time course that captured progenitor-like cell types. The authors compared genetically identical lines in different cell types to identify molecular signatures that could predict differentiation efficiency into neuronal cells (Jerber *et al*. 2021). However, genetic diversity was limited in these human cell studies, and they lacked the sample sizes necessary to map trans eQTL interactions among genes that drive neural lineage specification.

Powerful mouse resources including the Diversity Outbred (DO) heterogeneous stock combine genetic variation from multiple parent strains in a randomized breeding design that minimizes population stratification and ensures balanced allele frequencies across the genome, providing optimal power for genetic mapping studies (Churchill *et al*. 2012). Recently, we applied a systems genetics approach to mESCs derived from DO mice to infer the complex gene regulatory network and genetic interactions that underlie naïve pluripotency (Skelly *et al*. 2020; Aydin *et al*. 2023). Specifically, we profiled chromatin accessibility, gene expression, and protein abundance in a large panel of DO mESCs and mapped thousands of QTLs that affected chromatin accessibility (caQTLs), transcript abundance (eQTLs), and protein abundance (pQTL) (Skelly *et al*. 2020; Aydin *et al*. 2023). In this study, we extend our systems genetics approach to characterize variation in transcript abundance and identify cell type–specific and conserved eQTLs in mouse NPCs derived from many of the same DO mESC lines used in our previous studies (Skelly *et al*. 2020; Aydin *et al*. 2023). In line with our findings in DO mESCs, we observe high expression variation among the DO mNPCs. Moreover, despite large expression differences overall between the mESC and mNPC cell types, we observed high covariation in mESC and mNPC transcriptomes from the same DO donors. Genetic mapping identified thousands of significant local and distant eQTLs in mNPCs, and a comparison to mESC eQTL showed that local eQTLs were more likely than distant eQTLs to be shared between cell types. Most distant eQTLs, by contrast, were uniquely detected in 1 cell type, including 3 mNPC-specific eQTL hotspots on chromosomes (Chrs) 1, 10, and 11. Targets of the Chr 1 hotspot were enriched for roles in chromosome segregation and the spindle assembly checkpoint (SAC). Cross-cell-type mediation and partial correlation analysis identified 2 potential mediator genes of this hotspot, one expressed in the mNPCs (*Rnf152*) and the other earlier in the pluripotent mESCs (*Pign*). Both *Rnf152* and *Pign* have been implicated in cell cycle regulation with *Pign* having a direct role in the SAC (Okamoto *et al*. 2020; Teye *et al*. 2021), suggesting that these genes drive variation in cell cycle regulation in DO mNPCs. More broadly, our study demonstrates the power of applying a systems genetics approach across timepoints in a developmental trajectory, which allowed us to map eQTLs and link gene expression variation in 1 cell type to a causal variant affecting expression of a regulatory gene in a progenitor cell.

## Materials and methods

### Neural differentiation of DO mESCs

DO ES cell lines were produced as previously described (Skelly *et al*. 2020). Neural stem cell lines were produced by differentiation of ES cell lines following previously established protocols (Pollard *et al*. 2006). Briefly, cryopreserved ES lines were thawed and carried for 3–6 passages prior to differentiation. ES cells were trypsinized, and 50–75 K cells were plated per well of a laminin-treated 12-well plate in NS Media with FGF2 and EGF (Pollard *et al*. 2006). The medium was changed daily for 8 days, then detached with accutase, washed with NS media, and replated into laminin-coated 6-well plates with 2 mL per well of NS media. Neural stem cells were expanded in clusters for 3 days and then serially expanded into 6-cm and then 10-cm dishes until subconfluent. NS cells were passaged for an additional 3 weeks and then cryopreserved. NS lines were screened positive for neural stem cell markers such as NESTIN and GLAST and negative for ES cell markers such as SSEA1. The lines were further validated by differentiation to terminal neural cell types such as astrocytes and neurons, as demonstrated by flow cytometry after cell type–specific staining.

### DO mNPC RNA-seq

Total RNA was isolated from each of 186 DO mNPC lines and quantitated by paired-end (PE) RNA sequencing. Briefly, for each mNPC line, 1 15-cm dish of cells was grown to near confluence, washed 3× with PBS, and mechanically harvested to yield 10 M cells. About 100k cells from each frozen cell pellet were used for RNA sequencing. Next, total RNA was extracted using the Quick-RNA 96 well format kit (Zymo Research) with in-column DNase treatment. Sequencing libraries were prepared by Akesogen using the TruSeq Stranded mRNA HT kit (Illumina, cat. no. 20020595) and included ribosomal RNA reduction and poly-A selection, enzymatic fragmentation, cDNA synthesis from random hexamer priming, adaptor ligation, and PCR amplification steps to generate indexed, stranded mRNA-seq libraries. Libraries were checked for quality and quantitated with the Agilent Bioanalyzer, and samples that failed QC were repeated starting from the cryovial stage. Finally, pooled libraries were sequenced on the NextSeq platform (Illumina) using the NextSeq 500/550 High Output v2 150-cycle kits (Illumina, cat. no. FC-404-2002). To minimize technical variation, samples were randomly assigned to lanes prior to sample processing steps, barcoded, and multiplexed at 16 samples per flow cell, yielding 6M-55M 2 × 75 bp PE reads per sample.

We aligned PE 75-bp reads with bowtie v1.1.2 (Langmead *et al*. 2009) to a pooled “8-way” transcriptome containing strain-specific isoform sequences from all 8 DO founder strains as previously described (Skelly *et al*. 2020). In order to identify and correct sample mix-ups, we inferred sample genotypes from the RNA-seq data using GBRS v0.1.6 (Choi *et al*. 2020) and compared GBRS-derived genotypes to our DNA genotypes obtained from GigaMUGA arrays. Correlations between genotypes inferred from GBRS and GigaMUGA for the same sample were typically on the order of 0.8–0.9, whereas for different samples they were <0.5. We resolved 17 sample mix-ups for DO mNPC lines showing an incongruent genotype. For resolving multimapping reads and quantifying transcript- and gene-level expression counts, we utilized EMASE as implemented in GBRS v0.1.6 (Raghupathy *et al*. 2018; Choi *et al*. 2020). We filtered genes with a median transcripts per million value <0.5 or 0 value (i.e. not expressed) in more than half of the samples. To account for differences in library size, we normalized gene-level counts to the upper-quartile value then applied the ComBAT function from R/sva package to remove batch effects caused by library preparation (Johnson *et al*. 2007). We transformed normalized and batch-corrected values to rank normal scores using rankZ normalization (Gatti *et al*. 2014). Gene annotations such as MGI symbol, gene location, and gene biotype were added using v84 of the Ensembl database.

### DO mESC RNA-seq

Raw RNA-seq data for DO mESC samples were retrieved from ArrayExpress (E-MTAB-7728) and analyzed same as to the DO mNPC RNA-seq data.

### Statistical analysis

All analyses and figures were generated with the R statistical programming language (R Core Team 2023). Unless otherwise stated, R/tidyr package was used for data processing, R/ggplot2 for plotting, and R/pheatmap for heatmap plots.

### Functional enrichment analysis

We performed functional enrichment analysis using the “gost” function in the gProfiler2 package (Raudvere *et al*. 2019; Kolberg *et al*. 2020a, 2020b) by controlling the version using “set_base_url(set_base_url(‘https://biit.cs.ut.ee/gprofiler_archive3/e102_eg49_p15/’))” in R using an appropriate universal background on a case-by-case basis and “fdr” option for *P*-value correction. For example, we used the list of all genes that are expressed in NPCs for enrichment analysis of the DO mNPC Chr 1 hotspot target eQTL. For gene set enrichment analysis, we utilized the R/fgsea package (Korotkevich *et al*. 2021) with gene sets that belong to the Gene Ontology Biological Processes subcategory (Ashburner *et al*. 2000; The Gene Ontology Consortium *et al*. 2023) in the molecular signatures database (MSigDB) (Subramanian *et al*. 2005; Liberzon *et al*. 2011; Castanza *et al*. 2023) and 10,000 permutations.

### Differential expression analysis

Differential expression was calculated for the 12,095 genes expressed in both mESCs and mNPCs from the same DO donor (*n* = 127 lines total). In addition to the removal of the batch effects, sex effects were also removed before the comparison in both datasets using ComBAT as implemented in the R/sva package (Johnson *et al*. 2007). We compared gene expression using the Wilcoxon rank-sum test, implemented as the “wilcox.test” function in the R/rstatix package, and adjusted raw *P*-values to correct for multiple testing using the Benjamini–Hochberg (“BH”) method. The log2 fold change was calculated by taking the log2 of the ratio between the mean expression of each gene in mNPCs over mESCs.

### Correlation analysis

The correlation between the transcriptomes of genetically identical mESC and mNPC lines was calculated using the “rcorr” function with “type = ‘spearman’” option from the R/Hmisc package using the 11,671 autosomal genes expressed in both cell types for 127 cell lines. The “rcorr” function returns the Spearman correlation coefficients of all pairwise comparisons between the 127 cell lines, and this 127-by-127 matrix was subsetted to obtain the correlation values for the matched donor and mismatched donors distribution.

### QTL mapping

Heritability of each transcript was calculated using “est_herit()” function in R/qtl2 package with chromosomal sex included as a covariate and the full kinship matrix (Broman *et al*. 2019). Genetic mapping was performed using the R/qtl2 package (Broman *et al*. 2019) with wrapper functions utilizing parallelization for efficient large-scale eQTL analysis in R/QTLretrievR (https://github.com/deweyhannah/QTLretrievR). Briefly, eQTLs were mapped with a linear mixed model—implemented as the “scan1'” function in R/qtl2—using the upper-quartile normalized, batch-corrected, rankZ-transformed gene expression values, with chromosomal sex included as an additive covariate and the Leave One Chromosome Out (LOCO) option used for kinship correction (Yang *et al*. 2014). To estimate genome-wide significance, we permuted genotypes 1,000 times while maintaining the relationship between the phenotype and covariates. For each permutation, we retained the maximum LOD score to generate a null distribution for the test statistic (Churchill and Doerge 1994). To calculate thresholds for eQTL, we repeated this permutation strategy for all transcripts and estimated a significance cutoff at LOD > 7.5 (alpha = 0.05) and a suggestive cutoff at LOD > 6. False discovery rates (FDRs) (*q*-values) were determined for each permutation-derived *P*-value with R/qvalue software, using the bootstrap method to estimate pi0 and the default lambda tuning parameters (Storey *et al*. 2004), and our LOD threshold of 7.5 corresponds to an FDR of 0.075 and LOD threshold of 6 corresponds to an FDR of 0.16. We call a QTL “local” if the QTL peak is within ±10 Mbp to the midpoint of its corresponding gene and “distal” if otherwise.

Founder allele effects were estimated as best linear unbiased predictors at the QTL peak using “scan1blup” function in R/qtl2 package. To identify overlaps with significant NPC eQTL, we used a relaxed threshold of LOD > 5 for ESC eQTL. They were classified as shared if the eQTL peaks were within +/−5 Mb of each other and the correlation between haplotype effects was significant (adjusted *P*-value < 0.1).

For hotspot calling, we first identified distal eQTL that reach genome-wide permutation-based threshold (*P* < 0.05; LOD 7.5). Next, we applied a sliding window method to identify hotspots as described in (Skelly *et al*. 2020). Briefly, we counted the number of distal eQTL within 1cM windows (0.25 cM shift) across the genome and selected the top 0.5% of bins with the most distant eQTL (0.5% bin threshold 9 distant eQTLs). Final coordinates for each hotspot were determined using the Bioconductor package “GenomicRanges” to merge adjacent bins into a single region (Lawrence *et al*. 2013). Each hotspot contains >20 significant distant eQTL (>150 suggestive, LOD > 6) and shows distinct allelic effect patterns.

### Partial correlation analysis

We used the “pcor” function from the R/ppcor package (Kim 2015) to calculate the partial correlation between the expression of the mediator gene and the first principal component of the expression of genes with the target eQTL (Eigen-Chr1) on Chr 1. To control for the genetic effects in the partial correlation analysis, we classified the genotypes at the Eigen-Chr1 QTL peak based on their ancestry from the 8 DO founder strains. Given the 4:4 split at the Eigen-Chr1 QTL peak, if the cell lines showed homozygous ancestry from any of the strains in the B6/129/NOC/CAST group, they were classified as “Ref,” if they showed homozygous ancestry from any of the AJ/NZO/PWK/WSB strain group, they were classified as “Alt,” and if they showed ancestry from both groups, they were classified as “Het.” We calculated the partial correlation for all the candidate mediators within ± 10 Mb of the Eigen-Chr1 QTL peak using both gene expression from DO mESCs and mNPCs.

### Mediation analysis

We performed mediation analysis to identify candidate causal genes in mNPC eQTL hotspots. Mediation analysis was performed using the R/intermediate package (https://github.com/simecek/intermediate) by regressing target eQTL on the expression of a candidate mediator in the QTL and adjusting for covariates. We applied the “double-lod-diff” method to reduce the effects of missing values. For mediation of QTL with the matching cell type, we used the full sample set, e.g. eQTL mediation by NPC transcripts was done using all the 186 samples. Mediation across cell types was performed with mESC expression data from the common set of 127 DO donors. To assess the significance of a LOD drop, we mediated the QTL against all expressed genes genome-wide in that cell type to establish a null distribution of LOD score drops, converted the recorded LOD scores to normal scores, and checked whether the score fell below 4 SDs from the mean of the null distribution. Mediators were further filtered to narrow down top candidates to include genes with midpoints that are found within ± 10Mb of the QTL peak.

## Results

### Gene expression in genetically diverse mNPCs is highly variable and covaries with gene expression in mESCs

**Fig. 1.**
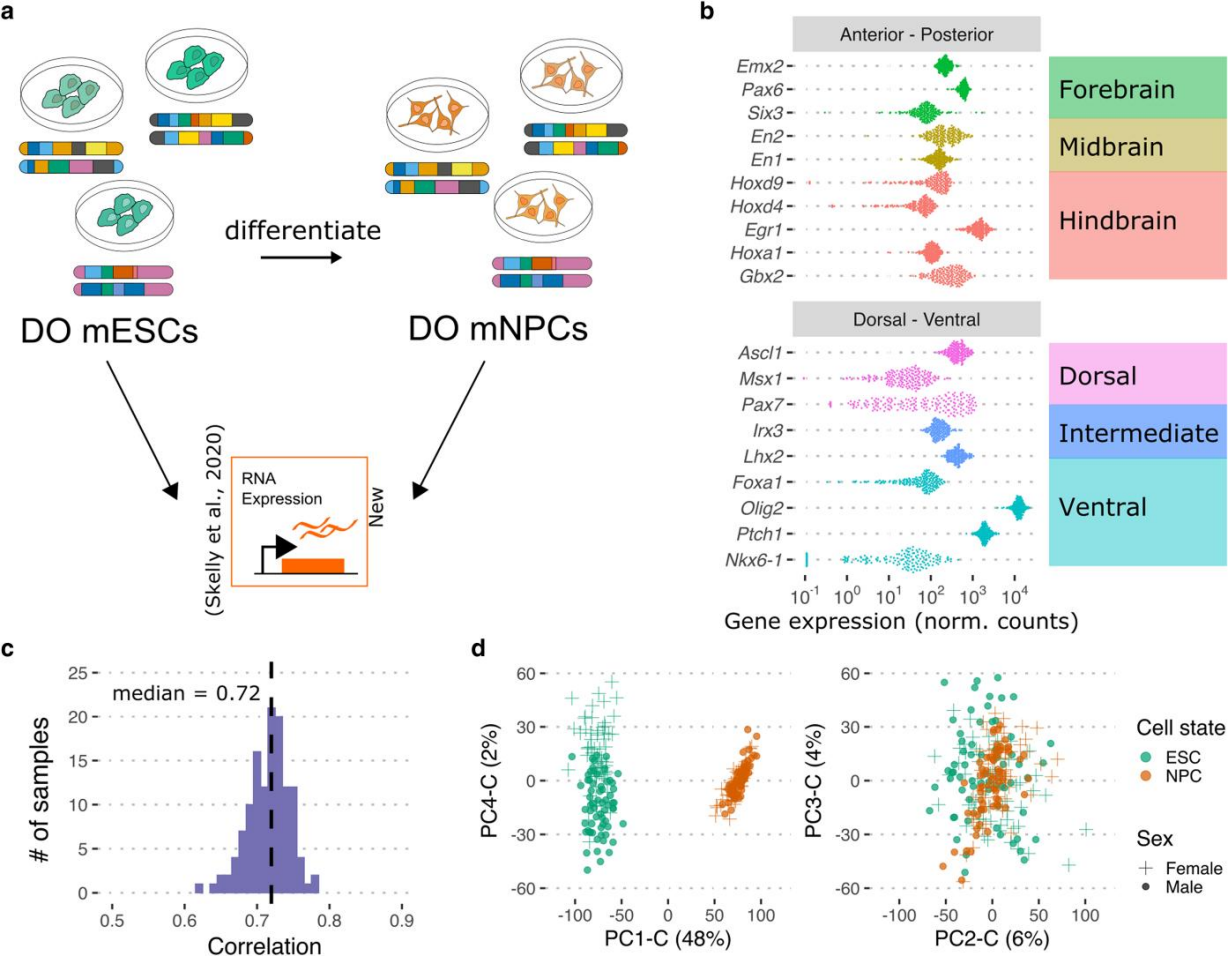
Variation in DO mNPC transcriptome and covariation to DO mESCs. a) Experimental overview of the data. b) Expression of neural lineage identity marker genes. The dots represent normalized gene expression counts for individual DO mNPC lines. c) Histogram of correlation coefficients between the ESC and NPC transcriptomes of 127 samples. d) PCA results of the combined ESC, NPC transcriptomes of 127 samples and 12,095 genes.

To better understand how the abundant genetic variation segregating in the DO population affects gene expression during neural differentiation, we differentiated 186 DO mESC lines to mNPCs (refer to diagram in Supplementary Fig. 1a and Materials and methods) and quantified the transcript abundance of 14,163 genes by RNA-seq (Fig. 1a). Correlations in transcript abundance between mNPC lines ranged from 0.6 to 0.99 (median = 0.97), and we performed principal component analysis (PCA) to identify the genes and pathways that are most variable across the DO mNPC lines. The first principal component (PC1-N, Supplementary Fig. 1b) explained 14% of expression variation among DO mNPCs, and functional enrichment analysis showed that PC1-N driver genes were overrepresented for those involved in the cell cycle, mRNA processing, translation, response to leukemia inhibitory factor (LIF), in utero embryonic development, and targets of transcription factors with roles in neural differentiation such as *Otx2* (Vernay *et al*. 2005) (Supplementary Table 1). A closer look at the cell cycle–related processes revealed genes with roles in almost all of the cell cycle checkpoints, underscoring the proliferative nature of mNPCs under expansion culture conditions. DO mNPC lines expressed markers of all 3 anterior–posterior (AP) identities at similar median levels albeit with different levels of variability, with some lines showing very low levels of *Hoxd9* and *Hoxd4* expression (Fig. 1b). The expression of dorsal–ventral identity marker genes was more variable; specifically, *Olig2* and *Ptch1* expression was higher in all cell lines, while *Msx1*, *Pax7*, *Foxa1,* and *Nkx6-1* showed much higher variability in expression than other marker genes (Fig. 1b). Together, these expression patterns suggest that DO mNPCs have not adopted a specific AP identity and, although they contain a strong ventral influence, they have the potential to differentiate into both dorsal and ventral neural cell types.

Next, we sought to better understand how transcriptional variation in DO mNPC lines compared with variation in their progenitor pluripotent DO mESC lines. We previously characterized gene expression in mESCs from the same DO donor for 127 of the mNPC lines in the current study (Skelly *et al*. 2020). Over 12,000 genes were expressed in both cell types, and we observed significant covariation between the mESC and mNPC transcriptomes from individual donors (median intraline NPC-ESC correlation = 0.72, Wilcoxon *P* < 5e−04; Fig. 1c and Supplementary Fig. 1c), likely indicating a large role for genetic background in conferring expression variability. Similarly, we observed high concordance between the mean and variance in transcript abundance for genes expressed in both cell types, with a correlation of 0.77 (*P* < 2.2e−16) and 0.74 (*P* < 2.2e−16), respectively (Supplementary Fig. 1, d and e).

To discern the sources of expression variation that were unique to mNPCs or shared with mESCs, we performed PCA on the combined gene expression counts from both cell types (PCA-C). Cell type appeared to be the primary driver of expression variation in the combined dataset (PC1-C, 48%; Fig. 1d), despite the high within-line covariation of expression in mNPCs and mESCs. Functional enrichment analysis of PC1-C implicated ubiquitin-mediated protein degradation, nervous system development, signaling pathways regulating pluripotency, and cellular differentiation as the major drivers of gene expression variation between the mESC and mNPC lines (Supplementary Table 2). Of note, PC1-C showed high agreement with PC1-N values (cor = −0.9) for the 127 DO mNPC lines included in both analyses. However, we find little overlap in the genes contributing to PC1-N and PC1-C, with only 39 genes shared among 709 driver genes for PC1-N and 605 driver genes for PC1-C. While PC1-C clearly corresponded to cell-type expression differences, no other PC in the combined dataset appeared to distinguish between cell types; indeed, PC2-C and PC3-C did not show any separation, exhibiting a range of values that spanned both cell types, and likely capturing variation common to both (Fig. 1d). Drivers of PC2-C and PC3-C were enriched for genes involved in essential cellular processes including the mitotic cell cycle, mitochondrial cell function, mRNA processing, membrane lipid metabolism, and chromatin organization (Supplementary Table 2). PC4-C appears to differentiate mESC lines by their chromosomal sex, which was previously observed and attributed to the 2 active X chromosomes in the pluripotent mESC state (Skelly *et al*. 2020; Aydin *et al*. 2023). This strong sex effect is not present in the mNPCs where the second X chromosome is known to be inactivated (Fig. 1d). Variance decomposition analysis confirms this difference in sex effect on gene expression in mESCs and mNPCs (Supplementary Fig. 1f).

To better characterize biological processes that were differentially regulated across the cell types, we performed differential gene expression analysis. We identified 1,409 upregulated and 773 downregulated genes in mNPCs relative to mESCs (adjusted *P* < 0.05, abs(log2 fold change) > 2). Genes upregulated in mNPCs were enriched for biological processes including nervous system development and pathways involved in neural differentiation such as WNT signaling. Conversely, genes downregulated in mNPCs were enriched for biological processes including ribosome biogenesis and pathways regulating pluripotency such as response to LIF (Supplementary Table 3), confirming the PCA results.

### Genetic architecture of the mNPC transcriptome

**Fig. 2.**
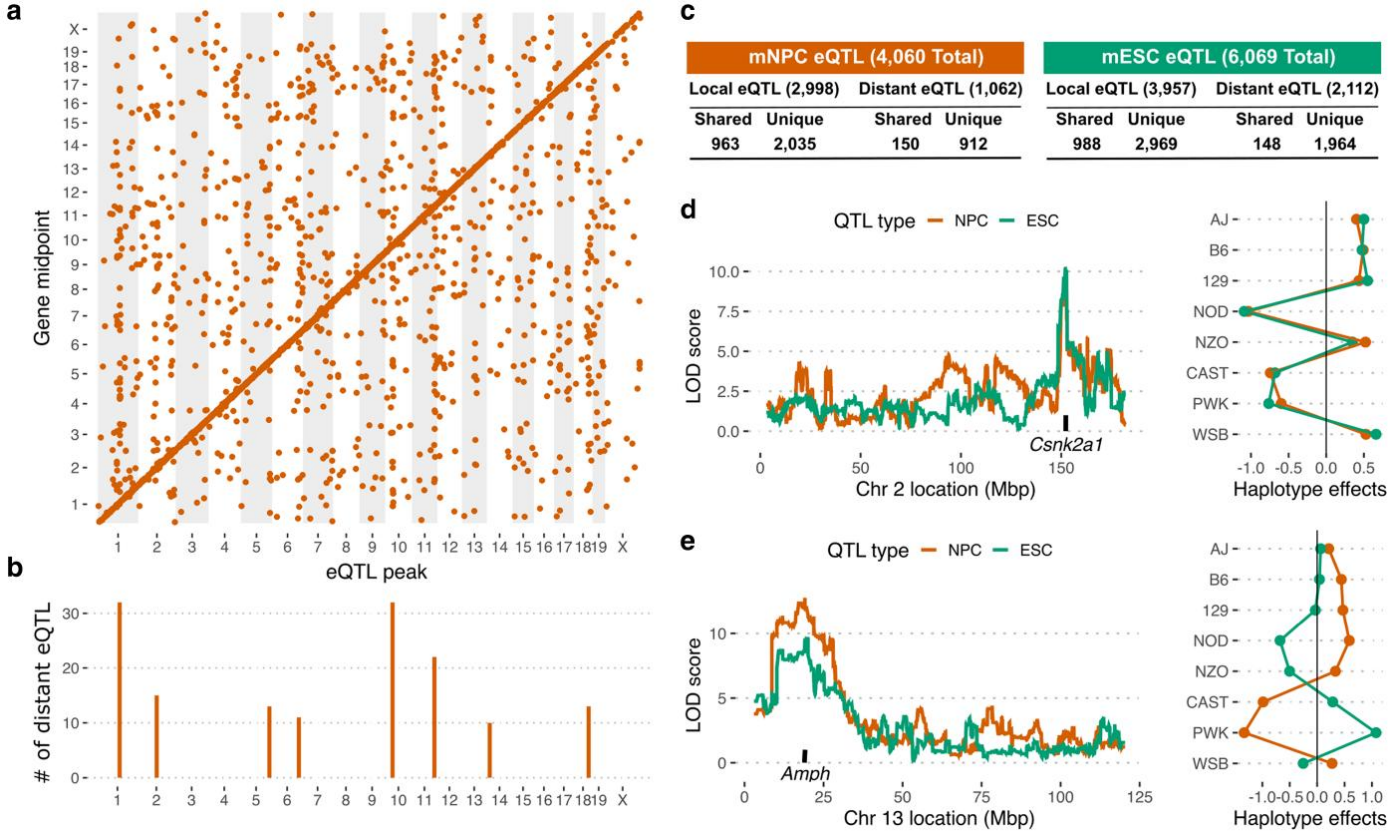
Genetic architecture of DO mNPC transcriptomes. a) Map of DO mNPC eQTL containing 4,060 significant peaks (LOD > 7.5) from 3,806 unique genes. b) eQTL hotspots identified across the genome. c) Comparison of DO mNPC and mESC eQTL results. d) eQTL scan for Csnk2a1 gene on the left and the haplotype effects at the eQTL peak on Chr 2 on the right. e) eQTL scan for Amph gene on the left and the haplotype effects at the eQTL peak on Chr 13 on the right.

To further characterize the sources of interline expression differences in mNPCs, we estimated narrow sense heritability. We estimated the transcript abundance of over 90% of expressed genes in mNPCs to be heritable (median *h*^2^ = 0.23), confirming that genetic diversity among DO mNPCs is a major driver of transcriptome variation. Genetic mapping identified 4,060 significant eQTL (LOD > 7.5, alpha = 0.05, FDR = 0.075) in mNPCs, with almost one-third of expressed transcripts having 1 or more significant eQTL (Supplementary Table 4, Fig. 2a). The majority of eQTLs are local (2,998/4,060), meaning the genetic variant impacting the abundance of the transcript is within close proximity (±10 Mb) to the gene itself. The remaining quarter are distant eQTL (1,062/4,060), where the peak is further away from the gene or on a different chromosome and likely influences the target gene's expression indirectly through direct effects on the expression or function of another “mediator” gene. We used a sliding window approach to assess genomic enrichment of distant eQTL (see Materials and methods) and observed that many distant eQTL colocalized to eQTL “hotspots,” similar to previous eQTL analyses (Brem and Kruglyak 2005; Chesler *et al*. 2005; Cheung *et al*. 2005; Keurentjes *et al*. 2007; Brynedal *et al*. 2017; Yao *et al*. 2017; Skelly *et al*. 2020; Kolberg *et al*. 2020a; Ben-David *et al*. 2021) . We detected 3 such eQTL hotspots on Chrs 1, 10, and 11 that were unique to mNPCs (Fig. 2b and Supplementary Fig. 2d and Table 5). Interestingly, we also mapped a significant QTL for PC1-N values (LOD > 7.11, alpha = 0.1) to the same hotspot on Chr 1, suggesting that this hotspot is a causal driver of global transcriptional variation in mNPCs (Supplementary Fig. 3a).

Next, we compared our mNPC eQTL map to the mESC eQTL map from our previous study (Skelly *et al*. 2020) (Fig. 2c and Supplementary Fig. 2a–c). Among the 2,998 local mNPC eQTL, 32% (963/2,998) are also detected in mESCs with significantly correlated founder allele effects, while the other 68% (2,035/2,998) are either not mapped in mESCs (1,385/2,035) or affect expression of a gene in mNPCs that is not expressed in mESCs (650/2,035). Among the 1,062 distant mNPC eQTL, only 14% (150/1,062) are also detected in mESCs with correlated founder allele effects, while the other 86% (912/1,062) are either not mapped in mESCs (782/912) or affect expression of a gene in mNPCs that is not expressed in mESCs (130/912). Thus, a higher proportion of local mNPC eQTL are shared with mESCs, while distant mNPC eQTL are predominately unique to that cell type. An example of a shared local eQTL with highly positively correlated founder allele effects is for *Csnk2a1* (Fig. 2d), a gene known to regulate various cellular processes including WNT signaling, cell cycle progression, apoptosis, and transcription, and mutations in this gene have recently been linked to neurodevelopmental abnormalities in humans (Okur *et al*. 2016). We also map shared local eQTL with opposing allele effects on gene expression in mNPCs and mESCs. Although much fewer in number (22/963), one such example is *Amph* (Fig. 2e), a gene involved in synaptic vesicle endocytosis where loss of expression leads to cognitive deficits in mouse models (Paolo *et al*. 2002; Zhang *et al*. 2021). Finally, we note that the target genes of unique mNPC eQTL are enriched for roles in cell–cell signaling and nervous system development (Supplementary Table 6).

Compared to DO mNPCs, we mapped nearly a third more significant eQTL (6,069) in DO mESCs (Skelly *et al*. 2020), with a similar majority of mESC eQTL being local (3,957 local; 2,112 distant). As in mNPCs, we observed that distant eQTLs in mESCs cluster to hotspots in the genome; however, the location and target genes of eQTL hotspots differ between mNPCs and mESCs, with mESC hotspots localizing to Chrs 3, 4, 10, and 15 (Supplementary Fig. 2d) (Skelly *et al*. 2020). Moreover, we see a similar breakdown of shared and unique loci among the local and distant eQTL in mESCs (Fig. 2c), specifically that local mESC eQTL are more likely to be shared with mNPCs than distant mESC eQTL (25% of local mESC eQTL shared; 7% of distant mESC eQTL shared).

### Cross-cell-type mediation analysis uncovers the influence of genetic variation across cell types

To characterize the eQTL hotspots uniquely observed in DO mNPCs, we performed functional enrichment analysis to identify affected pathways and mediation analysis to identify candidate regulatory genes in the hotspot regions. For these analyses, we relaxed our inclusion criteria to include all target genes that mapped with a suggestive eQTL (LOD > 6, FDR = 0.16) in the 3 hotspot regions on Chrs 1, 10, and 11. Targets of the Chr1 hotspot were significantly enriched for genes involved in mRNA processing, DNA repair, chromatin organization, protein degradation, and cell cycle (adjusted *P*-value < 0.01; Supplementary Table 7). Genes involved in protein processing in the ER were significantly overrepresented for the hotspot on Chr 10, while the Chr 11 hotspot did not show any significant functional overrepresentation. Mediation analysis failed to identify strong candidate genes underlying the Chrs 10 and 11 eQTL, suggesting that these hotspots may stem from genetic variant(s) affecting abundance of an RNA species not profiled (e.g. miRNA) in the current study, a posttranscriptional modification of RNA, function or stability of a protein, or any mechanism that does affect the transcript abundance of the mediator gene. We excluded these loci from further analysis.

**Fig. 3.**
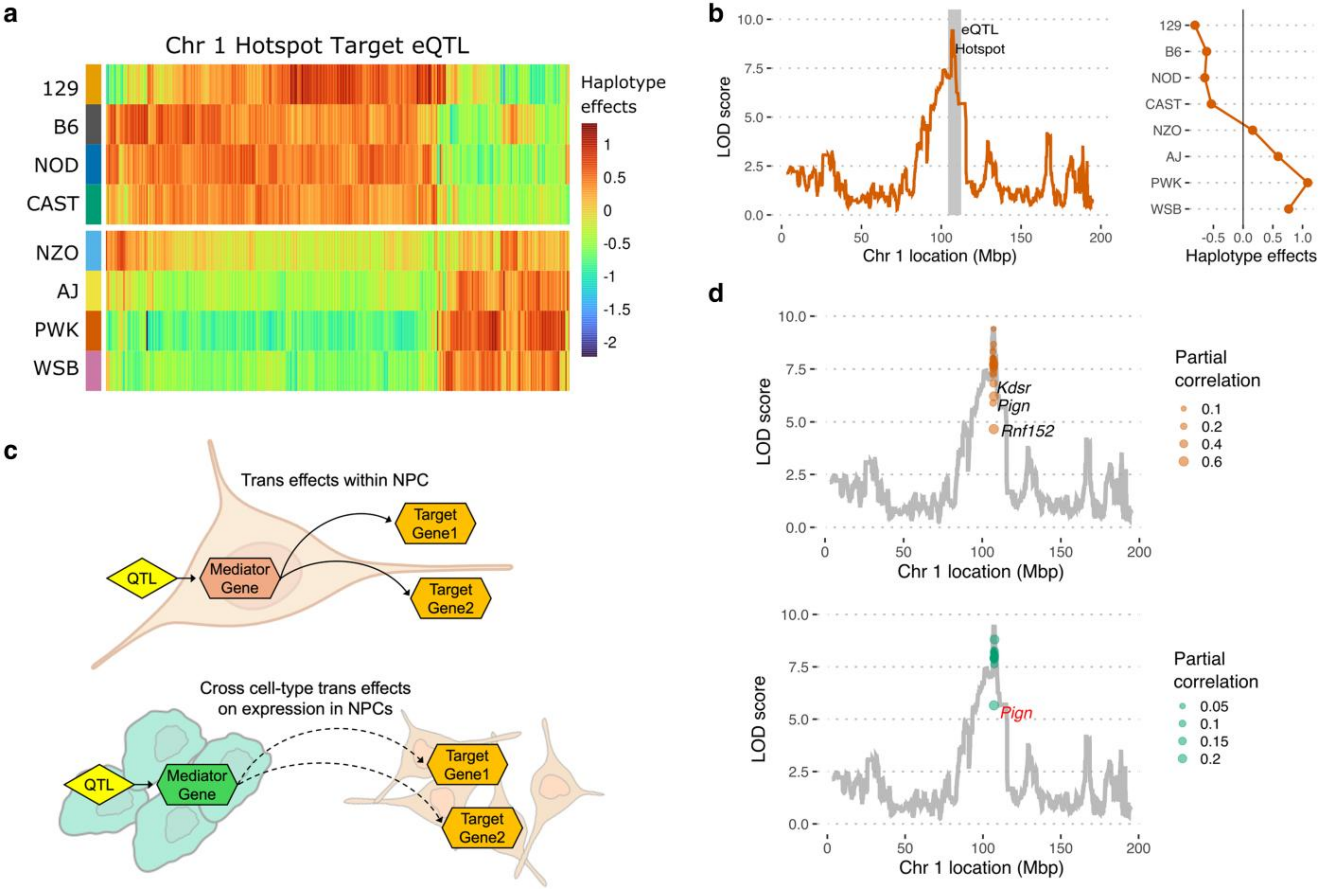
Details of the Chr 1 eQTL hotspot. a) Heatmap of founder haplotype effects at the eQTL peaks of the target eQTL (LOD >6, *n* = 322) within the Chr 1 hotspot. b) QTL scan of PC1-T of the Chr 1 target eQTL on the left, founder haplotype effects at the PC1-T QTL peak on the right. c) Cartoon depicting mediation analysis using ESC and NPC expression to identify potential regulators of an eQTL hotspot. d) PC1-T QTL scan is plotted overlaid with the new LOD scores obtained after mediation with NPC genes (above) and ESC genes (below) in the region.

Measurement error—including read mapping errors in RNA-seq datasets—can adversely affect individual gene expression estimates and introduce noise into eQTL mapping. Dimensionality reduction techniques like PCA are an effective way to summarize the main component of expression variation among eQTL hotspot target genes to infer founder allele effects and identify candidate mediator genes. Accordingly, we performed PCA on the transcript abundance of hotspot target genes and mapped QTL for the eigengene value (Eigen-Chr1). As expected, Eigen-Chr1 mapped with a significant QTL to the Chr 1 hotspot and exhibited founder haplotype effects similar to the observed individual target gene eQTL. Specifically, DO mNPC lines that inherited the Chr 1 hotspot region from B6, 129, NOD, or CAST founder strains showed similar expression of the eQTL target genes, while those lines that inherited this locus from AJ, NZO, PWK, or WSB showed a different pattern of gene expression (Fig. 3, a and b). Of note, the PC1-N Chr 1 QTL also showed the same 4:4 split in founder haplotype effects (Supplementary Fig. 3a). PC1-N values showed high agreement to Eigen-Chr1 (cor = −0.99), which likely reflects the significant overlap between the individual gene targets of the Chr 1 hotspot (overlap *n* = 193/322) and the gene drivers of PC1-N (overlap *n* = 193/709, hypergeometric *P* = 1e-86).

Given the large physical distance between eQTL hotspots and their target genes, it is generally assumed that the trans effects of these variants are conferred via direct proximal effects (i.e. cis effects) on the expression or function of a regulatory gene in the hotspot region. In cases where the causal genetic variant alters the expression level of the regulatory gene, mediation analysis can be a powerful tool to identify this “mediator” gene (Chick *et al*. 2016; Skelly *et al*. 2020). Importantly, an eQTL hotspot in 1 cell type (e.g. mNPCs) could stem from a genetic variant(s) that influences the expression of a regulatory gene in that same cell type *or* in a progenitor stem cell (e.g. mESCs). We performed mediation analysis on the Eigen-Chr1 values to identify potential mediators of the Chr 1 hotspot target genes, searching first in the mNPC expression data and then in the mESC expression data (Fig. 3c). Initially, we used a standard regression model to identify this potential local regulator within the Chr 1 hotspot (Chick *et al*. 2016). To gain more insight into the relationship between candidate mediators and the Chr 1 hotspot, we also performed partial correlation analysis—assessing the correlation of expression levels between candidate mediators and target genes after accounting for the observed effect of the distant eQTL—with the expectation that a true causal mediator should still co-vary in expression with the target genes even in the absence of the observed QTL effect. We calculated the partial correlation between Eigen-Chr1 and the expression of candidate mediators after accounting for the genetic ancestry at the QTL peak.

Mediation analysis with the mNPC expression data identified *Rnf152* as the best candidate mNPC mediator in the Chr 1 hotspot. That is, regressing out *Rnf152* expression in mNPCs caused the largest LOD drop in the most target eQTL and the Eigen-Chr1 QTL (Fig. 3d, Supplementary Fig. 3b). None of the candidate mediators, including *Rnf152*, were statistically significant as assessed by genome-wide scaling, but *Rnf152* showed the highest partial correlation to Eigen-Chr1 after regressing out the QTL effect (correlation = 0.6, adjusted *P* = 3.5e-12). *Rnf152* encodes a ubiquitin ligase residing in the lysosome; it is reported to be involved in mammalian target of rapamycin signaling and has been shown to regulate autophagy and cell proliferation (Deng *et al*. 2015; Okamoto *et al*. 2020). *Rnf152* has a local mNPC eQTL with a split in founder allele effects similar to the eQTL hotspot, consistent with its role as a Chr 1 hotspot mediator. (Supplementary Fig. 3c).

Next, we expanded our mediation analysis across cell types to identify potential mediators of the mNPC Chr 1 hotspot in the mESC expression data. We identified *Pign* as the best candidate mESC mediator for the mNPC Chr 1 hotspot, causing the largest LOD drops in the most target eQTL and the Eigen-Chr1 QTL. Further, mESC *Pign* expression showed the highest partial correlation to Eigen-Chr1 after regressing out the Chr 1 eQTL effect (correlation = 0.3, adjusted *P* = 0.1) (Fig. 3d, Supplementary Fig. 3b). The best mNPC mediator, *Rnf152*, was found to be expressed at very low levels in mESCs and did not pass our filtering threshold—consistent with previous reports (Lienert *et al*. 2011). *Pign* encodes for an enzyme involved in glycosylphosphatidylinositol anchor biosynthesis and more recently was linked to cell cycle regulation through interaction with the SAC complex (Teye *et al*. 2021). In DO mESCs and mNPCs, *Pign* has a significant local eQTL with effects that match the 4:4 founder allele split observed for the eQTL hotspot (Supplementary Fig. 3d). Importantly, target genes of the Chr 1 hotspot include those involved in proper chromosome segregation at mitosis such as *Mad2l1* (MAD2), a member of the SAC complex (Dobles *et al*. 2000).

## Discussion

Over the past 2 decades, genome-wide association studies in large human cohorts have identified thousands of common variants associated with variation in numerous complex traits and disease phenotypes (Abdellaoui *et al*. 2023). Many of these variants likely influence adult phenotypes through direct effects on gene expression or cell differentiation early in development, making them difficult to assay directly in humans (Maurano *et al*. 2012). In this study, we focused on neural differentiation and sought to better understand how genetic variation can influence gene expression using a large panel of mouse NPCs derived from the powerful DO heterogeneous stock. We analyzed interline variation in mNPC gene expression, performed eQTL mapping and mediation analysis, and compared our results to pluripotent mESC lines from the same donors. We identified genetic variation to be a significant driver of gene expression variability in DO mNPCs. Although they represent very different cell types and this difference is clearly evident in their transcriptomes, genetically identical mESC and mNPC lines showed high covariance in gene expression (median intraline correlation = 0.72) indeed, considerably higher agreement than we observed between transcript and protein abundance within genetically identical mESC lines (median intraline correlation = 0.36) (Aydin *et al*. 2023). Gene expression differences between cell types highlight known differentiation pathways, as genes involved in nervous system development and differentiation are upregulated in mNPCs while genes with roles in pluripotency maintenance are downregulated in mNPCs relative to mESCs. Meanwhile, genes involved in essential cellular functions such as the cell cycle, mitochondrial function, RNA processing, and membrane lipid metabolism were found to be variably expressed in both cell types. Given that mESCs and mNPCs are both self-renewing in culture, and given the dynamics and requirements of actively dividing cells, we were not surprised to observe high expression variability for genes involved in cell cycle regulation and cellular metabolism.

Expression QTL mapping added context to these expression patterns and uncovered thousands of genetic variants responsible for their expression variation. Over a quarter of significant eQTL detected in mNPCs were also mapped in mESCs and showed similar allele effects. Local eQTLs were more likely than distant eQTLs to be shared between cell types, and these shared eQTLs affected many genes with essential cellular functions. Population variation at these loci likely accounts for much of the high expression covariation observed in mNPCs and mESCs from the same DO donor. The remaining 3 quarters of mNPC eQTL appear unique to this cell type; of note, a similar proportion of mESC eQTLs were also cell type–specific (4,854/6,069 or 80%). Many of these cell type–specific distant eQTLs colocalize to genomic hotspots and likely play significant roles in cell lineage specification. For example, the previously identified Chr 15 eQTL hotspot regulating pluripotency maintenance is only observed in DO mESCs (Supplementary Fig. 2d) (Skelly *et al*. 2020; Aydin *et al*. 2023), while the hotspots on Chrs 1, 10, and 11 are only observed in DO mNPCs. Further, we found that genes involved in cell–cell signaling and nervous system development are significantly overrepresented among the unique distant eQTL in DO mNPCs. Together, these results highlight the abundance and widespread distribution of expression-modulating variants in genetically diverse populations, underscore a role for shared eQTL in driving interindividual variation within cell types, and provide further support for the importance of distant eQTL to lineage specification driving cell type–specific functions.

We identified 3 eQTL hotspots on Chrs 1, 10, and 11 with distinct genetic effects in DO mNPCs. Furthermore, mediation analysis helped us identify potential regulators for the hotspot on Chr 1. One of the top candidates, *Rnf152*, is a ubiquitin ligase that regulates autophagy. Although the drop in LOD scores obtained through mediation analysis did not achieve genome-wide significance for the target eQTL, *Rnf152* showed the highest partial correlation to the PC1 of the expression of target genes (i.e. Eigen-Chr1) when controlled for the founder strain ancestry at the QTL peak. Interestingly, the other candidate regulator, *Pign,* was identified from the ESC state. *Pign* was shown to regulate the SAC by forming a complex with the SAC proteins MAD1, MAD2, and the mitotic kinase MPS1 where loss of *Pign* expression led to an increase in errors in chromosome segregation and dysregulation of the SAC (Teye *et al*. 2021). In addition, *Pign* is linked to a variety of human disorders including some with neurological symptoms such as developmental delay, epilepsy, and seizures (Maydan *et al*. 2011). Although neither *Rnf152* nor *Pign* are transcription factors and are unlikely to directly regulate gene expression, they could be indirectly regulating the expression of downstream target genes through cellular signaling and influencing the progression of the cell cycle. *Rnf152* and *Pign* are separated by <200 kb on Chr 1, and we identified 4 candidate SNPs in the region that match the observed 4:4 strain allele effect (rs31971829, rs47676250, rs37516825, rs47457121) and may influence expression of 1 or both genes. Future experiments will seek to validate the causal variant(s) underlying this mNPC hotspot eQTL and establish the cell type of origin (i.e. mNPC or mESC) for its direct effects.

In this study, we obtain the first comprehensive look at the variation in the transcriptomes of a genetically diverse set of mNPCs. In addition, we characterized the influence of genetic variation across cell types using a large panel of genetically identical mESC and mNPC lines. Our experimental design allowed us to trace the impacts of genetic variation across 2 states in a developmental trajectory, which showed that primarily local genetic effects are shared between cell types. However, trans effects, even ones with large influences (e.g. *Lifr* allele in mESCs (Skelly *et al*. 2020)), are not conserved. Combined with the overrepresentation of neural development genes among those with unique distant eQTL in mNPCs, our results emphasize the specialized regulation of gene expression across cell types.

Finally, we acknowledge a number of study limitations that temper the strength of our conclusions. In the expression comparison of neural lineage marker genes in the DO mNPC lines, we note that our bulk RNA-seq data provides only a population average of transcript abundance and may mask heterogeneity among individual cells within each line. Cellular resolution of neural identity will require single-cell RNA-seq or similar profiles. In addition, although comparable in size to other published eQTL studies, our DO mNPC panel is not optimally powered to detect variants that confer only subtle effects. Given that essential regulatory genes may not tolerate large effect variants, we concede that our inability to detect small effect eQTL may cause us to miss important genetic interactions. Mediation analysis is a powerful tool but limited to cases where the causal variant(s) underlying the distant eQTL modulates the transcript abundance of the mediator gene. Distant eQTLs that stem from causal variants that influence posttranscriptional regulation, protein function or stability, or any other mechanism that does not impact transcript abundance of the mediator gene will be missed by mediation analysis. Similarly, partial correlation between genes does not necessarily indicate a direct regulatory or functional interaction.

## Supplementary Material

jkaf099_Supplementary_Data

## Data Availability

The raw and processed DO mNPC RNA-seq files are deposited to the Sequencing Read Archive (SRP553369) and Gene Expression Omnibus (GSE285231). Our upper-quartile normalized and batch-corrected gene expression matrix, the rankZ-transformed gene expression values, DO mNPC line genotype probabilities, and other objects used in eQTL mapping are deposited to figshare along with data analysis scripts (https://doi.org/10.6084/m9.figshare.28265894). We used previously published DO mESC gene expression matrix (ArrayExpress, E-MTAB-7728) and eQTL results (Skelly *et al*. 2020). Supplemental material available at G3 online.
