# Supplementary material for: Systems genetics reveals the influence of expression QTLs in mouse embryonic stem cells on transcriptional variation later in differentiated neural progenitor cells
Source: G3 (Bethesda). 2025 May 6;15(7):jkaf099. doi: 10.1093/g3journal/jkaf099 (PMC12239603; doi:10.1093/g3journal/jkaf099)
Supplement: jkaf099_Supplementary_Data [file jkaf099_supplementary_data.zip › FigureS1.pdf]

Supplemental Figures

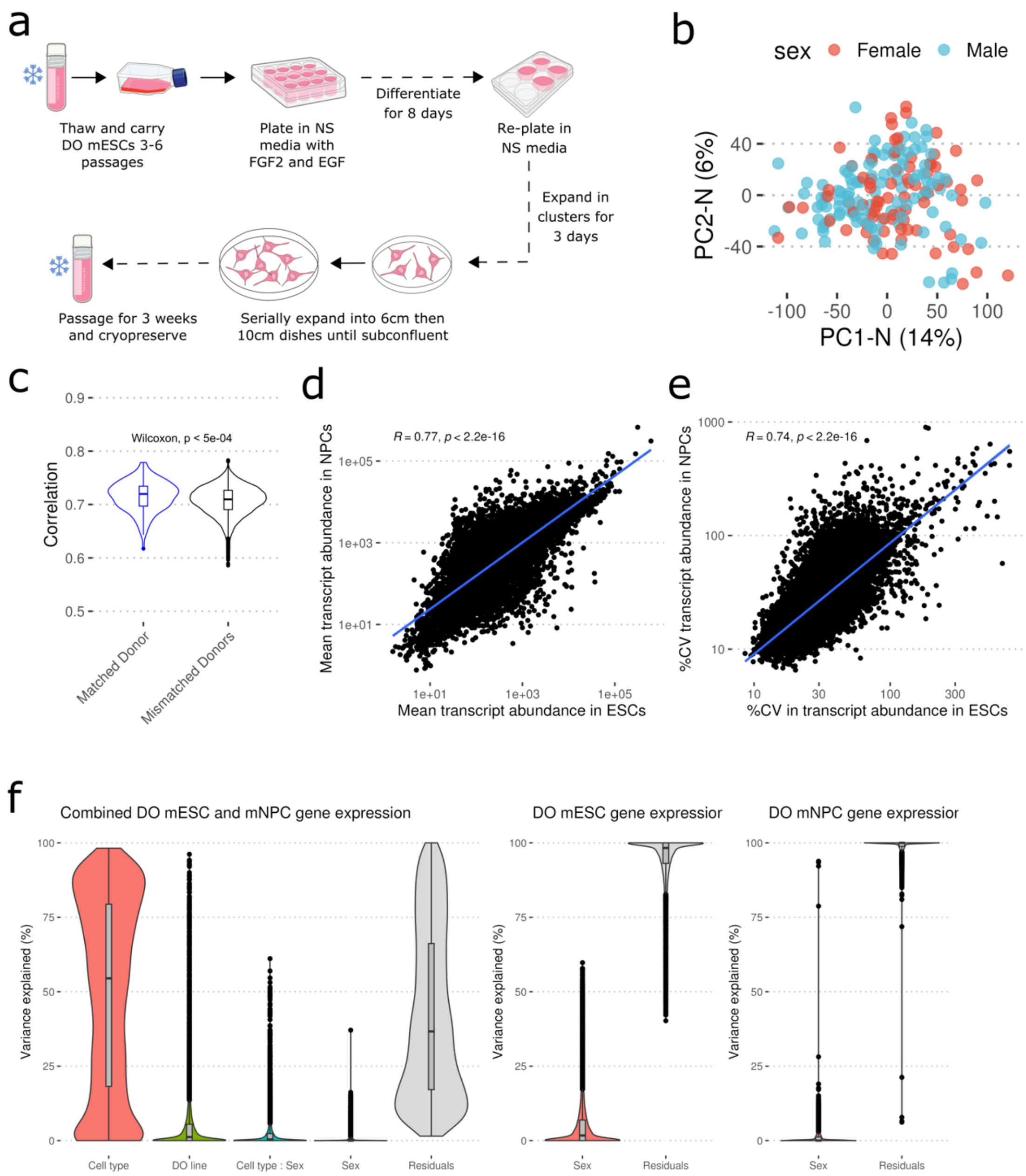

Figure S1. (a) Diagram depicting the main steps in the differentiation of DO mNPCs from DO mESCs. (b) Principal component analysis results of the DO mNPC data. (c) Genetically identical cell lines show significantly higher correlation than what is expected by chance between the ESC and NPC transcriptomes. Violin plots overlaid with boxplots depicting the distribution of Spearman correlation coefficients between the transcriptomes of 127 genetically identical mESC and mNPCs (blue) and the null distribution generated through 1000 permutations where the sample names are randomized (black). (d-e) Scatterplots showing mean and coefficient of variation (% CV) for transcript abundance for genes with measurements in both mESCs and mNPCs. (f) Violin plots overlaid with boxplots showing the variance partition analysis results in the combined and individual DO mESC, mNPC transcriptomes.
