# Supplementary material for: Systems genetics reveals the influence of expression QTLs in mouse embryonic stem cells on transcriptional variation later in differentiated neural progenitor cells
Source: G3 (Bethesda). 2025 May 6;15(7):jkaf099. doi: 10.1093/g3journal/jkaf099 (PMC12239603; doi:10.1093/g3journal/jkaf099)
Supplement: jkaf099_Supplementary_Data [file jkaf099_supplementary_data.zip › FigureS2.pdf]

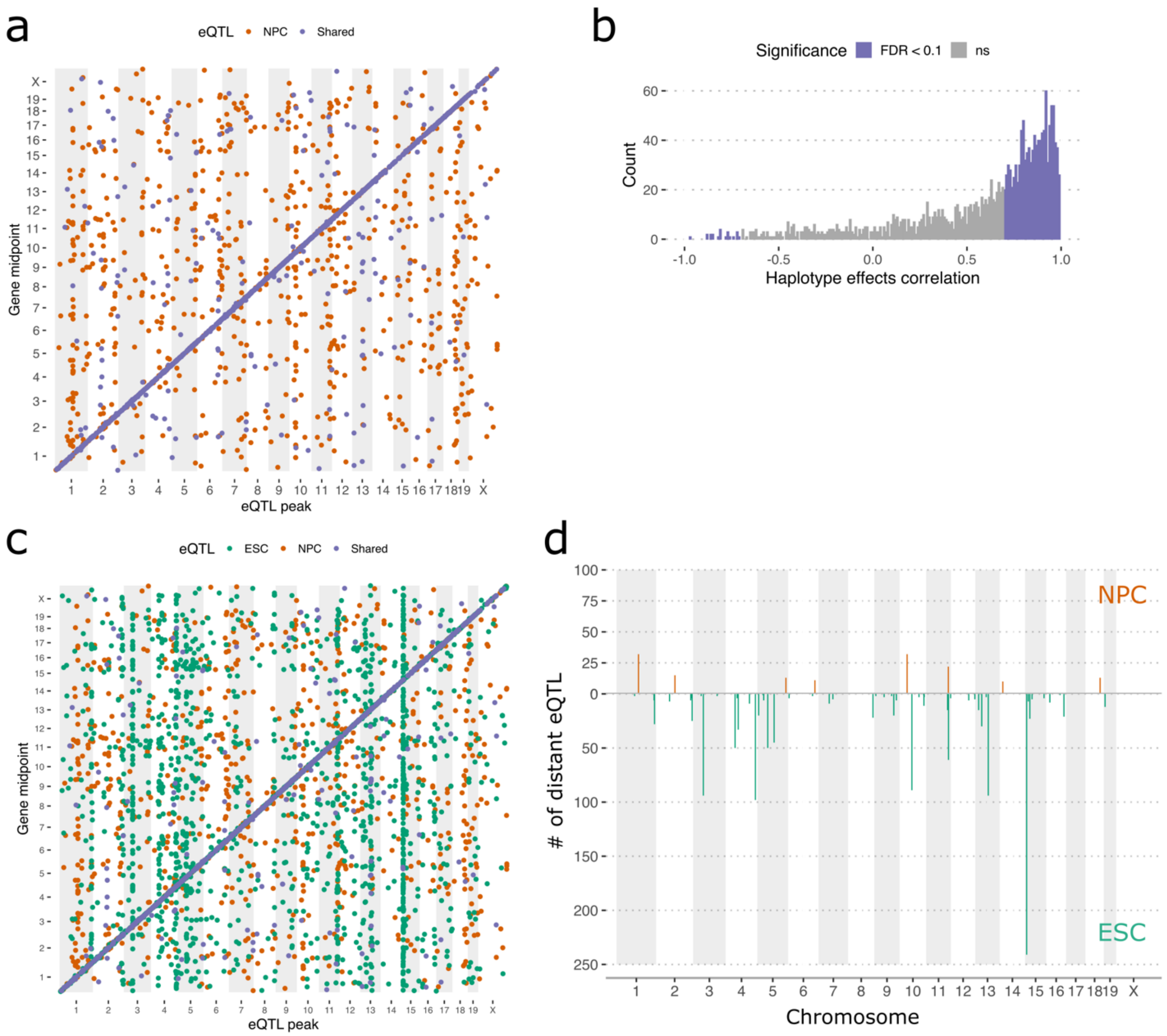

Figure S2. (a) Genetic mapping identifies 2,143 eQTL unique to NPCs and 2,529 eQTL shared between ESC and NPCs. The location of the eQTL is plotted on the x axis against the midpoint of the gene on the y axis. (b) Most co-mapping eQTL show high agreement in their haplotype effects. Histogram of pairwise correlation coefficients between inferred allele effects from eQTL from ESC and NPC scans for all genes with co-mapping QTLs. Bars are colored by significance of the correlation. (c) Compiled eQTL map showing the NPC-specific (orange) and shared (purple) eQTLs plotted in panel (a) with ESC-specific eQTLs (green) included. (d) Density plot showing the location of eQTL hotspots in DO NPCs and ESCs. Chromosome location is plotted on the x axis and the number of distal eQTL mapping to each region plotted on the y axis.
