# Supplementary material for: Systems genetics reveals the influence of expression QTLs in mouse embryonic stem cells on transcriptional variation later in differentiated neural progenitor cells
Source: G3 (Bethesda). 2025 May 6;15(7):jkaf099. doi: 10.1093/g3journal/jkaf099 (PMC12239603; doi:10.1093/g3journal/jkaf099)
Supplement: jkaf099_Supplementary_Data [file jkaf099_supplementary_data.zip › FigureS3.pdf]

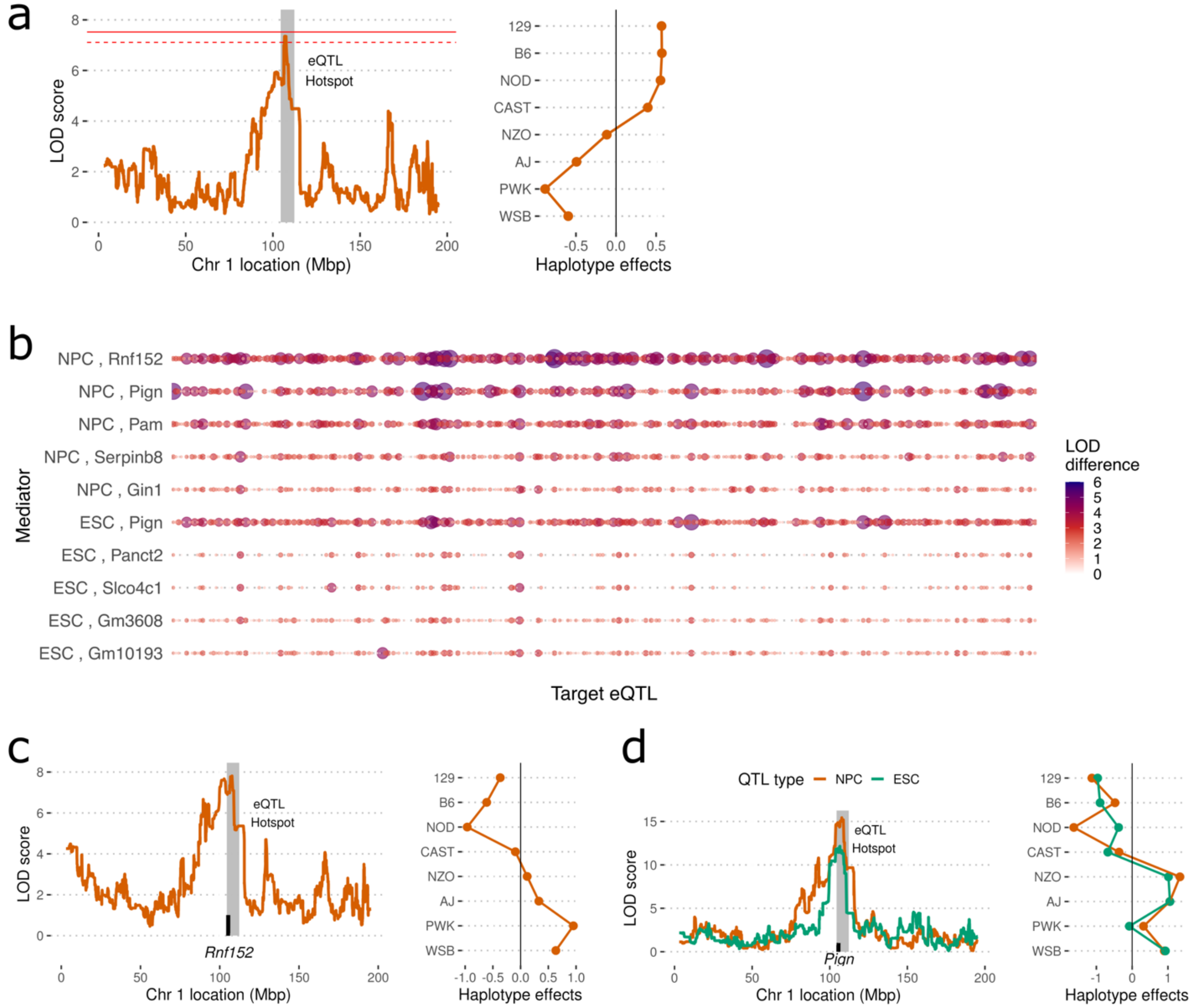

Figure S3. (a) Genetic mapping with PC1-N identifies a significant QTL on chromosome 1 with similar haplotype effects to the eQTL hotspot on chromosome 1. The red lines correspond to LOD thresholds for alpha = 0.05 (solid) and alpha = 0.1 (dashed). On the right, inferred haplotype effects at the QTL peak is plotted. (b) drops obtained from mediation analysis using NPC and ESC expression is plotted for the chromosome 1 target eQTL, showing results for the 5 best mediator genes within 10Mb of the eQTL hotspot. (c) Genome scan of *Rnf152* expression in NPCs show a significant local eQTL peak on chromosome 1 with inferred haplotype effects at the peak plotted on the right. (d) Genome scan of *Pign* expression in ESCs (green) and NPCs (orange) show a significant local eQTL peak on chromosome 1. The inferred haplotype effects at the peak are plotted on the right.
